# Antipsychotic Medications in Parkinson’s Disease Psychosis; A Systematic Review of Double-Blind, Randomised, Placebo-Controlled Trials

**DOI:** 10.3390/healthcare14050698

**Published:** 2026-03-09

**Authors:** Christopher John McKeown, Alberto Salmoiraghi

**Affiliations:** 1 Wrexham Maelor Hospital, Betsi Cadwaladr University Health Board, Croesnewydd Road, Wrexham LL13 7TD, UK; alberto.salmoiraghi@wales.nhs.uk; 2 Medical School, Wrexham University, Mold Road, Wrexham LL11 2AW, UK; 3 Medical School, Bangor University, Bangor LL57 2DG, UK

**Keywords:** Parkinson’s, psychosis, antipsychotic, quetiapine, clozapine, olanzapine, pimavanserin

## Abstract

**Highlights:**

**Main findings**

- Clozapine shown to improve Parkinson’s disease psychosis (PDP) with a large effect size despite low doses and does not appear to worsen motor function.
- Quetiapine has a poor evidence base for treatment of PDP, despite widespread use.

**Implications**

- Clozapine could be considered as a first line antipsychotic (AP) for the management of PDP if standard reductions in dopaminergic medications fail to achieve resolution of symptoms.
- APs with low Dopamine receptor 2 (D_2_) affinity may not appreciably worsen motor symptoms of Parkinson’s Disease.

**Abstract:**

Background: Psychosis is a common neuropsychiatric symptom associated with Parkinson’s disease (PD), with prevalence rates of up to 75% over the course of the disease. Parkinson’s disease psychosis (PDP) is associated with increased morbidity, caregiver burden, depression, poorer quality of life and progression of dementia. It has also been shown to be a strong predictive factor for long-term care placement, and results in up to 71% increase in risk of mortality compared with PD patients free from psychotic symptoms. Use of APs for PDP is common, with up to 35% of PD patients prescribed at least one AP within 7 years of PD diagnosis. Methods: Four electronic databases (Ovid MEDLINE, Embase, PsycINFO, PubMed) were systematically searched for double-blind, randomised, placebo-controlled clinical trials for the use of APs in the treatment of PDP and their effects on PD motor symptoms, according to PRISMA guidelines. Results: Eleven studies from ten publications were identified and included in this review. Four studies investigated quetiapine, three investigated olanzapine, two investigated clozapine and a further two investigated pimavanserin. Quetiapine showed no significant improvement for PDP over placebo in three of the four studies, with both olanzapine studies also showing no improvement. Olanzapine studies also showed significant motor worsening compared to placebo. Clozapine significantly improved psychosis compared with placebo in both studies, with large effect sizes in primary outcome measures; (−0.82, 95% CI −1.37 to −0.26), −0.89 (95% CI −1.42 to −0.36). Pimavanserin also showed significant improvement (−0.48, 95% CI −0.77 to −0.18). Quetiapine, clozapine and pimavanserin showed no significant worsening in motor scores compared with placebo groups. Conclusions: Data from the studies included in this review suggest that the use of quetiapine for the management of PDP may not be evidence based. Clozapine may improve PDP symptoms with low doses however significant side-effects may limit usability. The findings from this review support the use of clozapine as an alternative AP for the management of PDP when clinically appropriate.

## 1. Introduction

Parkinson’s Disease (PD) is a progressive, neurodegenerative disorder characterised by bradykinesia, rigidity, resting tremor and postural instability, resulting from neuronal cell loss within the Substantia Nigra Pars Compacta and subsequent dopamine (DA) depletion [1]. It is the second most common neurodegenerative disorder globally, affecting over 6 million individuals [2], with the fastest growing prevalence; incident cases exceeding one million annually as of 2017 [3,4]. Although diagnostic criteria focus on motor dysfunction, PD also presents with other non-motor symptoms, which may occur up to 20 years prior to clinical motor manifestation [5]. This prodromal phase of PD can be characterised by autonomic disturbance, olfactory dysfunction, sleep disorders and psychiatric symptoms [6,7,8,9,10].

Psychotic symptoms may occur in up to 75% of PD patients during the course of the disease process, depending on psychosis definitions and rating scales [11,12,13,14], and are a significant burden of disease. Parkinson’s disease psychosis (PDP) is associated with increased morbidity, depression, poorer quality of life [15], caregiver burden [16], development and progression of dementia [17,18] and has been shown to be a strong predictive factor for long-term care placement [19]. Recent studies show those diagnosed with PDP at an increased risk of hospitalisation [20] and up to 71% increase in risk of mortality compared with PD patients free from psychotic symptoms [21].

Dopaminergic medications have long been suggested as the main causative factor leading to PDP [22,23], with all PD medication types associated with psychotic symptoms [24]. Changes in dopaminergic therapy have been observed to precipitate PDP, with improvements in psychosis seen following reduction or cessation [25], however psychotic symptoms have been reported in up to 42% of drug-naive PD patients [26,27], suggesting that their occurrence may be intrinsically linked with the disease process itself rather than solely attributable to dopaminergic therapy used to manage motor dysfunction.

When psychotic symptoms are distressing, intolerable or fail to improve with adjustment of dopaminergic therapy, AP treatment may be required [28]. The use of AP medications in patients with PD is widespread, with 12% of patients prescribed an AP within the first 12 months of PD diagnosis and 35% of patients within seven years [29,30], and has been associated with worsened motor symptoms of PD and increased mortality [31].

Currently, only clozapine has a UK licence for the treatment of PDP, however accounts for <2% of AP prescribing. Quetiapine accounts for 49–66% of AP prescriptions in PDP [29,32], despite no clear advantage over placebo in a previous meta-analysis of double-blind, randomised placebo-controlled trials [33].

Previous systematic reviews and meta-analyses investigating APs in PDP have been completed, with a number limited to atypical class [34,35,36,37,38,39] or single drug reviews [33,40]. A single meta-analysis [34] completed in 2015 includes wide search terms for APs, and specifies only double blind, randomised, placebo-controlled trials for inclusion, and further systematic reviews and network meta-analyses have been completed including any APs whilst also including comparator [37] and open label studies [36,39]. Details of relevant studies are summarised on Table 1.

This review aims to provide an up-to-date analysis of double-blind, randomised, placebo-controlled trials investigating the treatment of PDP with no restriction by AP class, searching all AP medications that are in use or have been used, worldwide. The most recent systematic review with these strict inclusion criteria was published in 2015 [34] and this review serves as an appropriate update to the current body of literature. Preliminary findings from this review were presented in abstract and poster form at the Royal College of Psychiatrists International Congress, 2024. This manuscript has been substantially expanded and updated with recent literature searches, detailed methodology including risk of bias assessment, reporting of dose titration schedules and how these may have impacted outcomes, with a focus on detailed narrative synthesis to help inform readers of the strengths and weaknesses of the included studies. A limited pooled meta-analysis was also performed to provide supporting quantitative evidence. This review aims to further inform clinical decision-making regarding AP choice, dosing and titration schedules in PDP, given ongoing uncertainty about the relative roles of commonly used agents in routine practice, where quetiapine often remains a first-line treatment.

## 2. Materials and Methods

### 2.1. Study Design

**Figure A1:**
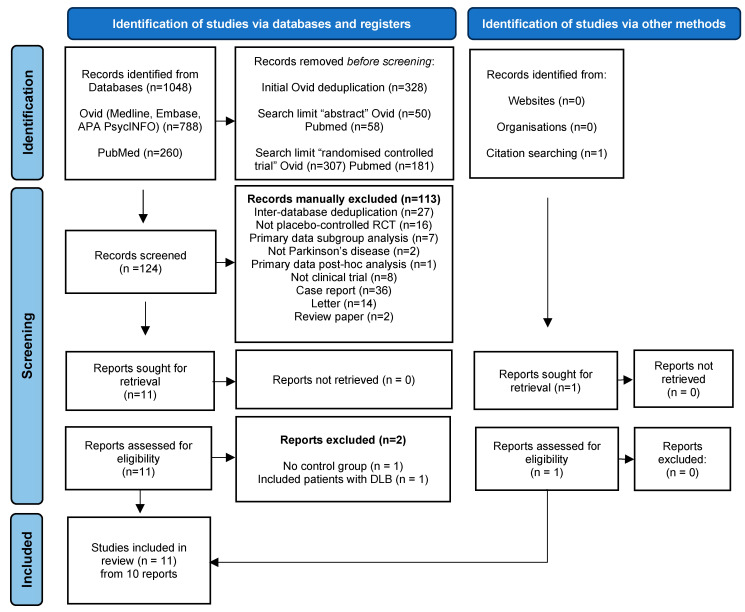
PRISMA 2020 flow diagram for database searches.

This systematic review was conducted with reference to the PRISMA (Preferred Reporting Items for Systematic Reviews and Meta-Analyses) 2020 statement guidelines (for PRISMA flow diagram, see Appendix A, Figure A1) [41]. The review protocol was developed a priori, however was not registered.

### 2.2. Objectives

The aims of this review are to identify, appraise and synthesise evidence from published, peer-reviewed double-blind, randomised, placebo-controlled trials investigating the efficacy and tolerability of APs in the management of PDP, with particular focus on motor outcomes.

### 2.3. Search Strategy

**Figure A2:**
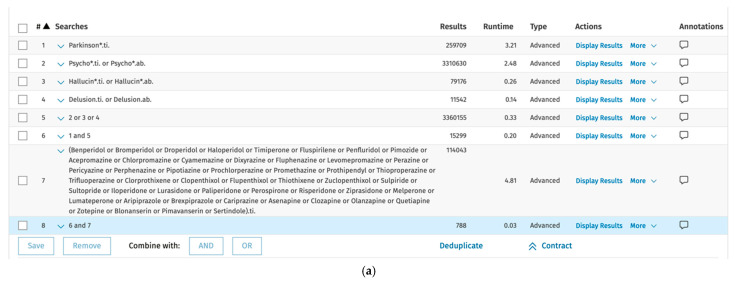

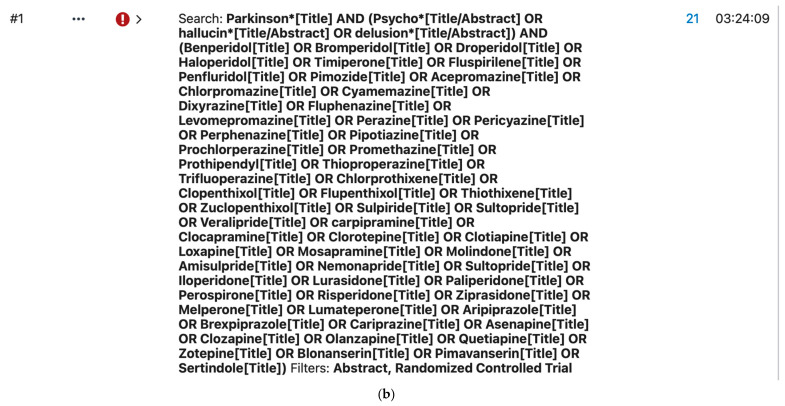
(**a**), Search strategy for Ovid (MEDLINE, Embase, PsycINFO). Search Limits “Abstract” and “Randomised Controlled Trial” applied to results following automated deduplication. (**b**), Search strategy for Pubmed. Search limits “Abstract” and Randomised Controlled Trial” applied to results. * is used for truncation in the search criteria-standard for electronic databases.

Searches were restricted to fully published and peer-reviewed papers: Electronic database searches were conducted via Ovid, including MEDLINE (1946 to 7 November 2025), Embase (1974 to 2025 Week 45), APA PsycINFO (1806 to Nov 2025 Week 1). Additional searches were performed in PubMed and Google Scholar to identify further relevant references (Nov 2025). Clinical trial registries, unpublished and non-peer reviewed sources were not systematically searched or included. Peer-review was considered an essential criterion for study inclusion, to ensure methodological scrutiny. The list of APs was derived by searching available literature and the online resources [42,43,44]. To maximise yield of relevant papers, selected keywords were truncated to account for differences in spelling and pluralisation and to cover a broader range of relevant related terms. Search strategy for each database can be found in Appendix A, Figure A2a,b.

### 2.4. Eligibility

Inclusion Criteria:

- Double-blind, randomised, placebo-controlled trials.
- Treatment arm using recognised AP medication.
- Participants with a clinical diagnosis of idiopathic PD and psychotic symptoms
- Studies reporting quantified measures of psychosis and motor outcomes using validated rating scales as either primary or secondary outcome measure.
- English-language, peer reviewed publications.

Exclusion Criteria:

- Comparator or open-label studies.
- Reviews, case reports, conference abstracts without full data or unpublished data.
- Studies including patients with diagnosed dementias such as dementia with Lewy bodies, unless data could be separated.

### 2.5. Outcomes of Interest

- Primary Outcome: Change in psychotic symptoms in PD, measured by validated instruments.
- Secondary outcome: Change in Motor symptoms, measured using validated instruments
- Adverse events and discontinuations reported.

### 2.6. Study Selection and Data Extraction

Titles and abstracts were screened independently by both authors. Full texts of potentially eligible publications were reviewed independently by both authors against inclusion criteria, with discrepancies resolved by discussion. Data were extracted from eligible publications by the lead author, using a modified Cochrane Data Collection Tool and are tabulated in Table 2 (trial characteristics; participant demographics, psychotic symptoms, entry criteria, PD diagnostic criteria, measures used, titration schedule) and Table 3 (descriptive results; psychosis outcomes, motor outcomes, dropouts and adverse events). Raw numerical data for included publications are included in Supplementary Materials.

### 2.7. Quality Assessment

**Figure A3:**
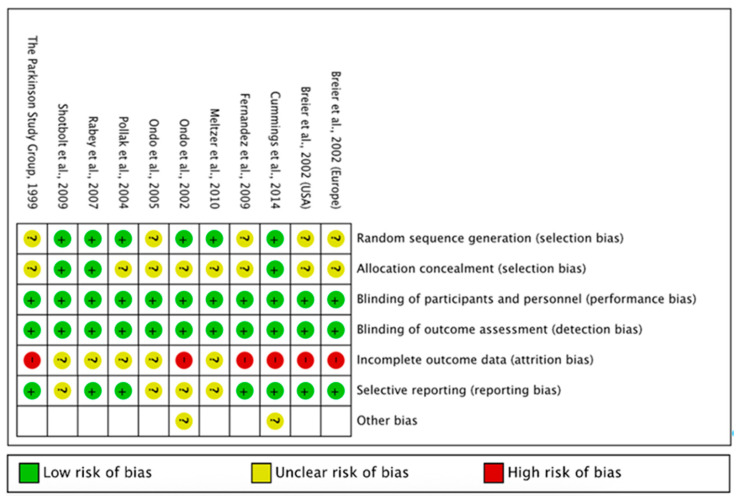
Risk of Bias assessment Summary Table [45,46,47,48,49,50,51,52,53,54].

Risk of Bias was assessed by the lead author using RoB 1 (integrated in RevMan 5.4 software) provided by the Cochrane collaboration [55]. Inclusion of studies was not dependent on outcome of risk of bias assessment. Assessment included reviews of selection bias (random sequence generation and allocation concealment), performance bias, detection bias, attrition bias and reporting bias. Domains were assessed as low, high or unclear risk of bias Results are summarised in Appendix A, Figure A3. Domains were judged following guidance in the Cochrane Handbook for Systematic Reviews of Interventions [56].

### 2.8. Data Synthesis

Due to the anticipated small number and heterogeneity of trials, a full pooled meta-analysis for all APs was not planned a priori. However, where trials of the same AP were comparable in outcome measures and appropriate data were available, limited inverse-variance random-effects meta-analyses were performed using RevMan 5.4 statistical software, provided by the Cochrane Collaboration [55]. A random-effects model was utilised for pooling of estimates to account for clinical and methodological heterogeneity, including differences in study duration and dosing strategies. Statistical heterogeneity was assessed using the I^2^ statistic. Where pooling was not appropriate due to heterogeneity in outcome measures or insufficient data, findings have been synthesised descriptively and grouped by AP to allow comparison of efficacy, motor effects and tolerability, as well as direction and magnitude of effect. Effect sizes were calculated where adequate data were available.

### 2.9. Reference Management

All references were managed in Mendeley Cite, and data extraction tables were prepared in Microsoft Word. Database search results were managed in Rayyan [57] for further deduplication and completion of manual screening.

## 3. Results

A total of 1048 publications were returned from database searches. Following initial deduplication “abstract” and “randomised controlled trial” filters were applied as part of the structured a priori search strategy. 124 papers remained and were manually screened to assess eligibility for inclusion. 113 were excluded (reasons provided in PRISMA flow diagram Appendix A, Figure A1). Full text review of eleven publications was completed. One included patients with Dementia with Lewy Bodies and was disregarded [58]. One lacked a control group and was also disregarded [59] as per pre-defined inclusion criteria. Reference lists of included publications were screened for further relevant papers, yielding one further study that met criteria for inclusion [49]. Ten papers comprising a total of eleven individual clinical studies fulfilled the criteria for inclusion, with a total of 645 patients: 129 from the four quetiapine studies, 137 from the two olanzapine studies, 120 from the two clozapine studies and 259 from the two pimavanserin studies. All included studies utilised single active intervention arm versus placebo; no adjustment for multi-armed designs was required. Where a publication reported two distinct randomised trials, these were extracted and analysed as two separate studies. Four investigated quetiapine [45,46,47,48], two olanzapine [49,50], two clozapine [51,52] and two pimavanserin [53,54]. Cognitive exclusion criteria varied between studies. One excluded patient with dementia [54], although no definition of dementia was provided. Unified Parkinson Disease Rating Scale (UPDRS) Part 1 “intellectual impairment” is however recorded in the results. Two excluded patients with either “significant cognitive impairment” or dementia, if unable to complete testing [48,51] without specifying how these patients were assessed. 

**Table 3:** Description of Study Results.

Author	Psychosis Outcomes	Motor Outcomes	Dropouts		Adverse Events	
			Active	Control	Active	Control
Shotbolt et al., 2009 (UK) [45] Quetiapine	No statistically significant changes in psychosis between groups as measured by NPI, Baylor scale and BPRS. NPI hallucination and delusion sub-scores showed no statistically significant changes between groups.	UPDRS III (motor) scores not significantly different between groups	Participants N = 11 5—Unspecified 3—Drowsiness 8—total	Participants N = 13 5—Unspecified 2—Drowsiness 1—Confusion 8—total	3 drowsiness	2 drowsiness 1 confusion
Rabey et al., 2007 (Israel) [46] Quetiapine	No significant changes in psychosis from baseline and between both groups, as measured with BPRS and CGI-S. BPRS subgroup analysis of hallucinations/delusions showed no significant differences between groups.	UPDRS III (motor) scores not significantly different between groups	Participants N = 30 10- Lack of therapeutic response 3—Unspecified 2—Somnolence 15—total	Participants N = 28 9—Lack of therapeutic response 2—Unspecified 11 total	7—Somnolence 1—Symptomatic orthostatic hypotension 1—Headache 1—Syncope	2—Somnolence 1—Syncope
Ondo et al., 2005 (USA) [47] Quetiapine (2:1)	No significant changes in psychosis between groups as measured by BPRS and Baylor scale. BPRS item 12 subgroup analysis of hallucinations showed no differences between groups.	UPDRS II (Activities of daily living) + III (motor) scores + GDRS not significantly different between groups	Participants N = 21 2—Lack of effect and poor compliance 2—serious unrelated illness 4—total	Participants N = 10 2—serious unrelated illness resulting in death 2—total	9—Sedation 4—Subjective worsening of PD 10 individual AEs not specified	4—Sedation 10 individual AEs not specified
Fernandez et al., 2009 (USA) [48] Quetiapine	Statistically significant improvement in psychosis in Quetiapine group vs. placebo as measured by BPRS item 12 (Hallucinations) and CGI-S. Total BPRS non-significant between groups.	UPDRS III (motor) scores not significantly different between groups	Participants N = 8 2—Lack of therapeutic response 1—Drowsiness 1—Lost to follow up 4—total	Participants N = 8 1—Unspecified	1—Confusion 3—Drowsiness 1—Increased appetite 3—Loss of balance/worsened Parkinsonism 1—Nightmares	1—Bronchitis 1—Confusion 1—Drowsiness 1—Dry mouth 4—Dizziness/Syncope 1—Depression 1—Sore throat
Ondo et al., 2002 (USA) [49] Olanzapine (2:1)	No significant changes in psychosis between groups as measured by structured interview for hallucinations. UPDRS item 2 (Thought disorder) sub-item analysis did not show any statistically significant changes.	UPDRS III (motor) scores significantly worsened in active group compared with placebo. Decline driven by worsening of gait and bradykinesia subscales. Tapping scores significantly worsened. Unclear if this is between groups or baseline to final screening.	Participants N = 18 1—Prior to taking medication 1—Lack of therapeutic response 2—total	Participants N = 12 1—Lack of therapeutic response.	6—Worsening movement 3—Worse posture 2—Dysarthria 2—Oedema 2—Drooling 2—Weight gain 1—Nausea 1—Insomnia 1—Sedation 1—Perspiration 1—Agitation	1—Insomnia 1—Sedation 1—Leg Cramps 1—Light-headedness 1—Weakness 1—Tremor 12 patients specified to have AEs, but information only given for the above
Breier et al., 2002 (USA) [50] Olanzapine (1:1)	No significant changes in psychosis between groups as measured by positive symptoms cluster sub-score of BPRS. BPRS total, negative symptom cluster, hallucinations, CGI-S psychosis, NPI total, hallucinations, delusions all showed no significant difference between groups	UPDRS total, II (ADL), III (motor) scores significantly worsened in Olanzapine group compared with placebo. No significant difference in UPDRS tremor between groups	Participants N = 41 16—Unspecified	Participants N = 42 7—Unspecified	10—Extrapyramidal syndrome 10—Hallucinations 9—increased salivation	1—Extrapyramidal syndrome 2—Hallucinations 2—Increased salivation
Breier et al., 2002 (Europe) [50] Olanzapine (2:1)	No significant changes in psychosis between groups as measured by positive symptoms cluster sub-score of BPRS BPRS total, negative symptom cluster, hallucinations, CGI-S psychosis, NPI total, hallucinations, delusions all showed no significant difference between groups	UPDRS total, II (ADL), III (motor) scores significantly worsened in active group compared with placebo. No significant difference in UPDRS tremor item between groups	Participants N = 49 12—Unspecified	Participants N = 28 4—Unspecified	Not detailed	Not detailed
The Parkinson Study Group, 1999 (USA) [51] Clozapine	Statistically significant improvement in psychosis in Clozapine group compared with placebo as measured by BPRS, BPRS-M, CGI-S, SAPS.	UPDRS total, III (motor) scores not significantly different between groups. Statistically significant improvement in tremor for Clozapine group vs. placebo as measured by UPDRS.	Participants N = 30 1—Leukopenia 1—Myocardial Infarction 1—Sedation 3—total	Participants N = 30 2—Worsening psychiatric symptoms 1—Hospitalised for pneumonia 3—total	Not detailed	Not detailed
Pollak et al., 2004 (France) [52] Clozapine	Statistically significant improvement in psychosis in Clozapine group compared with placebo as measured by CGI-S and positive PANSS	No statistical worsening of motor scores between groups	Participants N = 32 1—Neutropenia 1—Fracture 2—total	Participants N = 28 1—Hypotension 1—Syncope 2—total	7—Worsening of PD 3—Sialorrhoea 17—Somnolence 1—Constipation 6—Postural Hypotension 5—Respiratory infection 2—Neutropenia 1—Seizures	1—Worsening of PD 2—Confusion 5—Somnolence 4—Nausea/vomiting 1—Constipation 4—Postural Hypotension 3—Respiratory infection 3—Condition aggravated 4—Syncope/malaise
Cummings et al., 2014 (USA, Canada) [53] Pimavanserin	Statistically significant improvement in psychosis in Pimavanserin group compared with placebo as measured by SAPS-PD SAPS Hallucinations ± Delusions sub items, CGI-S all showed statistically significant improvements for Pimavanserin over placebo.	UPDRS II, III + composite score all showed no statistically significant worsening of motor scores between groups.	Participants N = 105 6—Psychosis 1—Sepsis 1—Septic shock 1—did not receive dose 9—total	Participants N = 95 1—Sudden cardiac death 1—Unspecified 2—total	6—Nausea 7—Peripheral Oedema 14—Urinary tract infection 11—Fall 6—Confusional State 1—Headache 7—Hallucination	6—Nausea 3—Peripheral Oedema 11—Urinary tract infection 8—Fall 3—Confusional state 5—Headache 4—Hallucination
Meltzer et al., 2010 (USA) [54] Pimavanserin	No significant changes in psychosis between groups as measured by SAPS (Primary outcome measure) SAPS sub items for hallucinations + delusions all non-significant, with exception of Global rating of hallucinations and delusions; significant improvement vs. placebo.	UPDRS II, III + composite score all showed no statistically significant worsening of motor scores between groups.	Participants N = 29 1—Worsening hallucinations	Participants N = 31 1—Adverse event—Unspecified 1—“Disease Progression” 1—Other unspecified 3—total	3—Somnolence 3—Oedema 3—Elevated blood urea	5—Hallucinations 4—Dizziness 3—Fall 3—Headache 3—Confusional state 3—Hypotension

Abbreviations: ADL—Activities of Daily Living, AE—Adverse Event, BPRS—Brief Psychiatric Rating Scale, CGI-S—Clinical Global Impression Severity, GDRS—Goetz Dyskinesia Rating Scale, NPI –Neuropsychiatric Inventory, PANSS—Positive And Negative Syndrome Scale, PD—Parkinson’s Disease, PPRS—Parkinson’s Psychosis Rating Scale, SAPS—Scale for the Assessment of Positive Symptoms, SAPS-PD—Scale for the Assessment of Positive Symptoms in Parkinson’s Disease—UPDRS—Unified Parkinson’s Disease Rating Scale.

Formalised cognitive assessment using Mini-Mental State Examination (MMSE) [60] was undertaken in four papers as part of their exclusion criteria screening. Three used an inclusion cut off score of >20 [49,52,53] and one used a score of >21 [47]. The remaining three papers did not exclude patients on the basis of cognitive impairment or diagnosis of dementia, however, did complete MMSE testing [45,46,50].

Psychosis inclusion criteria differed between the included studies with regards to severity and instruments used to screen participants. One paper [54] utilised criteria developed for diagnosis of PDP, however the remaining nine used standard definitions of psychosis, not specific to PD. Three focused exclusively on hallucinations [47,48,49], with the remainder investigating both hallucinations and delusions.

Diagnostic criteria for Parkinson’s disease was not consistent for the included papers: five citing UK Brain Bank Criteria [45,46,48,52,53]; one utilising Diagnostic and Statistical Manual of Mental Disorders-IV criteria [50]; two papers diagnosed PD on either two or three “cardinal features” with no obvious exclusionary criteria for other parkinsonian presentations [49,51], and two did not report diagnostic criteria [47,54].

Risk of Bias assessment of the included studies found that both blinding of participants and personnel as well as blinding of outcome assessment were all considered to be low risk, however many studies did not describe how allocation concealment was maintained, leading to unclear risk of selection bias in eight of the included papers. Of particular note was the overall high risk of attrition bias in the papers, with no included study considered to be low risk. This may reduce confidence in the interpretation of treatment effects.

### Evidence Synthesis

Where pooling of data was undertaken for individual agents, a random-effects model was used to account for anticipated clinical and methodological heterogeneity. Due to the small number of included studies, statistical heterogeneity, as assessed by the I^2^ statistic, was interpreted cautiously.

Of the four quetiapine trials, three found that there was no statistically significant improvement in psychosis of any measure [45,46,47], with one paper showing significant improvements in Brief Psychiatric Rating Scale (BPRS) item II (hallucinations) and Clinical Global Impression-Severity (CGI-S) [48]. No significant worsening of motor symptoms was seen between groups. Meta-analysis was not undertaken for quetiapine due to incomplete data, omitting mean change difference (MD) ± Standard Deviation (SD) or Standard Error (SE).

**Figure 1:**
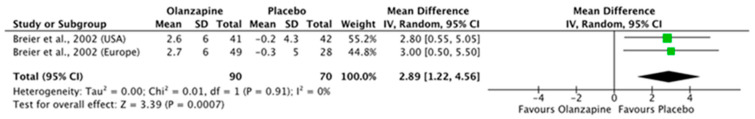
Random-effects model meta-analysis, olanzapine, motor (UPDRS) mean change difference [50].

Both olanzapine studies [49,50], demonstrated no statistically significant improvement in psychosis compared with placebo and showed significant worsening of motor function. Pooled analyses for psychosis and motor symptoms were completed for two olanzapine trials from one paper [50], with statistically significant worsening of motor symptoms observed on UPDRS mean change difference (Figure 1).

**Figure 2:**
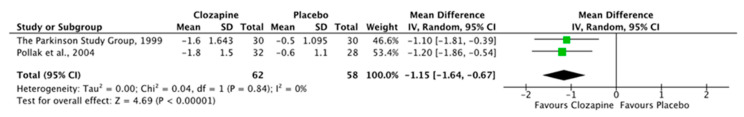
Random-effects model meta-analysis, clozapine, psychosis (CGI-S) mean change difference [51,52].

**Figure 3:**
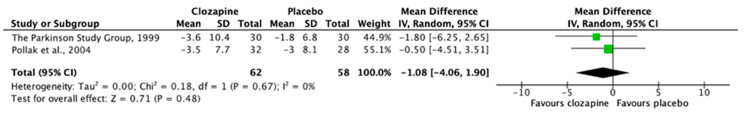
Random-effects model meta-analysis, clozapine, motor (UPDRS) mean change difference [51,52].

Both publications investigating clozapine [51,52] found significant improvements in measures of psychosis with a large effect size, and no statistically significant worsening of motor symptoms compared with placebo. These studies were sufficiently homogeneous in design and outcome measures to allow for pooling of data. Random-effects meta-analysis demonstrated a significant improvement in psychotic symptoms as measured by CGI-S scale mean change difference (Figure 2), with no statistically significant worsening of motor scores as measured by UPDRS (Figure 3).

Statistical heterogeneity within the analyses in this review was low, which may indicate consistent effects between pooled studies; however, this must be interpreted cautiously due to the small number of trials included for each agent.

Pimavanserin studies found no statistically significant worsening of motor symptoms, however differed in improvements in psychosis. One paper did not reach significance in primary efficacy outcome measures but found global rater-assessed measure of hallucinations and delusions to have improved significantly when compared with placebo [54]. One paper found significant improvement in psychosis using both primary and secondary efficacy measures when compared with placebo [53], however failed to reach a previously specified threshold for meaningful clinical relevance. Due to differences in the reporting of data between the two pimavanserin studies (one providing MD ± SE for both psychosis and motor symptoms, and one paper providing baseline and post treatment means ± SD, with no MD ± SD/SE), pooled analysis was not performed.

## 4. Discussion

Across the included studies, clozapine demonstrated the most consistent efficacy for treatment of PDP showing a large effect size in most measures of psychosis utilised by the papers, with only a single measure marginally missing the large effect threshold value of 0.8. Effect sizes are presented in Appendix A, Table A1, calculated using data provided by the included papers where available. Both studies’ CGI-S measure has shown a robust clinician rated treatment effect for PDP, which supports the significant treatment effect seen in both patient-rated BPRS and Positive and Negative Syndrome Scale (PANSS) positive.

UPDRS motor scores were not significantly different between groups in either paper, however a trend towards improvement was seen in those treated with clozapine. The Parkinson Study Group noted that there was an improvement in tremor in the clozapine group, not reported in Pollak et al., (2014) [52], which may account for this. Pollak et al. [52], report seven AEs of worsening Parkinsonism, however these did not require cessation of medication and were not reflected in overall motor scoring. Pollak et al., [52] also reported a high frequency of somnolence in the active group, which may reflect the relatively rapid titration of treatment when compared with the Parkinson Study Group, (1999) [51] paper. Both clozapine studies experienced at least one instance of neutropenia (NP) or leukopenia, which highlights the potential risk of inducing haematological abnormalities despite the low doses used. These findings broadly support the conclusions from the previous meta-analyses in Table 1, all of which showed consistent improvements in measures of psychosis from their included trials.

The studies using pimavanserin show a mixed picture with regards to efficacy, which is at odds with results from previous meta-analyses possibly due to the exclusion of two unpublished trials that are frequently included, as well as other trials including comparator studies that were not included in this review due to a priori inclusion/exclusion criteria. Both included pimavanserin studies in this review utilise the Scale for the Assessment of Positive Symptoms, Hallucinations and Delusions subsections (SAPS H + D) as a measure of psychosis, however Cummings et al., [53] showed a significant improvement, whilst Meltzer et al. [54], efficacy results fell short of significance. Mean SAPS H + D score reductions for pimavanserin groups in both studies appear similar, as do the mean score reductions in placebo groups. Considering the greater treatment effect seen by Meltzer [54], the lack of significance may be the result of a type 2 error, potentially due to relatively small sample size. Cummings et al., [53] utilised the Scale for the Assessment of Positive Symptoms—Parkinson’s Disease (SAPS-PD) as their primary outcome measure for psychosis, which is unique to this paper. It is unclear if this measure has been robustly validated as a measure of PDP, however the effect size seen is consistent with other measures of psychosis used in the study, which would suggest that this may be a valid measure of PDP. The authors have stated that an improvement of 1 on Clinical Global impression—Improvement (CGI-I) would be deemed to be a clinically meaningful improvement in PDP, which was associated with a change of 2.33 in SAPS-PD during its development [61]. Cummings et al., [53] did not observe an improvement of 1 on CGI-I, the benchmark for meaningful clinical improvement, despite SAPS-PD mean reduction of 3.06. Statistical significance of individual reported rates side effects for both studies have not been reported, however both studies appear to show an increased risk of peripheral oedema. Data from both studies show no statistically significant worsening of motor symptoms, which is consistent with pimavanserin’s very low D_2_ binding affinity (detailed in Table 4) resulting in negligible nigrostriatal DA blockade.

BPRS, CGI-S and UPDRS outcomes for the Breier et al., [50] studies support the findings of Ondo et al., (2002) [49] with olanzapine’s lack of efficacy for the treatment of PDP, as well as its negative impact on motor symptoms of PD. The AEs reported in the Ondo et al., USA study [47,49] add weight to the results, with significantly higher reported rates of extrapyramidal symptoms, hallucinations and increased salivation in the active group, however these differences were not seen in the European study. These results are in keeping with olanzapine’s comparatively high D_2_ receptor binding affinity, with previous studies [62] showing significant D_2_ receptor occupancy at low doses of olanzapine, comparable to mean dosages used in the included studies. This may explain PD patients’ particular sensitivity due to depleted nigrostriatal DA being subject to further blockade, worsening motor and extrapyramidal symptoms.

Three of the four studies utilising quetiapine included in this review showed no significant improvement in psychosis over placebo, however Fernandez et al., (2009) [48] reported significant improvement in two measures, with improvements in BPRS item 12 demonstrating a large effect size. The reasons for these significant results are unclear but may represent a type 1 error due to small sample size and method used for intention-to-treat analysis. All quetiapine studies showed no significant worsening of motor scores, again possibly due to quetiapine’s low D_2_ receptor binding affinity, again broadly supporting the findings of previously published literature.

**Table 4:** Receptor binding affinities (Ki, nM) of antipsychotics in this review.

Receptor	Clozapine ^1^	Quetiapine ^1^	Olanzapine ^1^	Pimavanserin ^2^
α_1_A	1.62	22	112	nr
α_2_A	37	3630	314	nr
α_2_C	6	28.85	28.9	nr
D_2_	157	379	34.23	nr
D_3_	269.1	340	47	nr
H_1_	1.13	6.9	2.19	nr
M_1_	6.17	489	2.5	nr
5-HT_1_A	123.7	394.2	2282	nr
5-HT_2_A	5.35	912	3.73	0.4
5-HT_2_C	9.44	1843	10.2	1.6
5-HT_6_	13.5	948.75	8.07	nr
5-HT_7_	17.95	307.6	105.2	nr

Abbreviations: α_1_A—adrenergic alpha receptor type 1A, α_2_A—adrenergic alpha receptor type 2A, α_2_C—adrenergic alpha receptor type 2C, D_2_—dopamine receptor type 2, D_3_—dopamine receptor type 3, H_1_—histamine receptor type 1. M_1_—muscarinic receptor type 1, nr—no response, 5-HT_1_A—serotonin receptor type 1A, 5-HT_2_A—serotonin receptor type 2A, 5-HT_2_C—serotonin receptor type 2C, 5-HT_6_—serotonin receptor type 6, 5-HT_7_—serotonin receptor type 7. Tabulated data are the equilibrium inhibitor constant values (Ki) expressed in nM. ^1^ Data sourced from cited literature [63] ^2^ Data sourced from cited literature [64].

The studies that have been included in this review suffered several methodological limitations that may affect their reliability. Power calculations were provided in only five of the eleven included studies [45,46,51,53,54], with only two of these recruiting the required numbers of participants [51,54]. A further significant limitation of the literature included in this review was incomplete outcome data, which limited statistical analysis, with only six studies providing adequate data required to calculate effect size appropriately (Appendix A, Table A1). Study design heterogeneity, including duration, dosages of medications, and titration schedules further complicates direct comparison, as does the use of differing scales for the assessment of psychosis. Entry criteria were also not consistent for all studies, with differing inclusion and exclusion thresholds for both cognitive function and psychosis. PDP has no formalised diagnostic criteria, however a National Institute of Neurological Disorders and Stroke/National Institute for Mental Health work group developed suggested criteria [65] which have been utilised by two of the included publications. Other publications in this review either conducted their studies prior to the publication or have used an alternative definition of PDP. This introduces further participant heterogeneity between the papers. Five studies were deemed to have an unclear risk of attrition bias, four of which utilised Last Observation Carried Forward for missing data. This method assumes stability of symptoms after dropout, and may introduce bias if attrition differs between groups. A further six studies were deemed high risk of attrition bias (see Appendix A, Figure A3). Incomplete follow up and potential bias reduce confidence in the reliability of calculated treatment effect estimates across studies.

The findings of this review are broadly consistent with the current body of evidence for the AP management of PDP (see Table 1), with clozapine demonstrating the most consistent efficacy signal and olanzapine showing poor efficacy alongside a propensity to worsen motor scores. Most recent reviews similarly find that although quetiapine does not appear to exacerbate motor symptoms in PD patients, its antipsychotic effect is comparable to placebo. The quetiapine trial demonstrating statistically significant improvements in CGI-S and the hallucinations subsection of BPRS (item 12) was associated with a 50% attrition rate in the active intervention arm, with non-completer data imputed using a linear model of completers. High attrition and small sample size reduces confidence in the robustness and reliability of these findings [48]. Pimavanserin is widely reported to produce statistically significant improvements in psychosis scores and remains a potential alternative to clozapine; however, the effect sizes observed for the included studies in this review were small to medium, and clinically meaningful improvements were not achieved in one of the two trials. This highlights that statistical significance within trial data does not necessarily translate into clinically meaningful benefit for patients.

## 5. Conclusions

Recent publications examining AP prescribing patterns indicate quetiapine accounts for approximately half of all prescriptions for the treatment of PDP. National Institute for Health and Care Excellence (NICE) guidelines recommend considering quetiapine in patients without cognitive impairment and offering clozapine where standard management is ineffective [28]. The findings from this systematic review suggest that, despite quetiapine’s low risk of worsening motor symptoms in PD, it has not demonstrated benefit over placebo for the treatment PDP in double-blind, randomised, placebo-controlled trials. Given the increased risk of mortality in patients prescribed APs, a clear therapeutic benefit should be seen to justify their use.

Currently available evidence does not support the use of quetiapine as a first-line AP for the treatment of PDP, particularly when other agents have demonstrated superiority to placebo, without appreciable worsening of motor scores. Notably, although efficacious for PDP, clozapine continues to carry a risk of significant adverse effects including NP even with the low dosages utilised in PDP trials. Owing to the potential for significant and life-threatening adverse effects, stringent haematological monitoring has historically been required for patients receiving clozapine. Perceived risk and monitoring burden may impact clinicians’ willingness to prescribe clozapine for PDP, potentially contributing to its relatively low utilisation despite evidence of efficacy and overall tolerability. This may in turn result in some patients’ psychosis remaining inadequately managed.

More recently, clozapine monitoring guidance in the European Union has changed to 12-weekly blood tests from the previous four-weekly if the patient has not developed NP within the first year of treatment, and then moving to yearly testing if no evidence of NP in the first two years. This is based upon large meta-analyses [66], retrospective cohort studies [67] and registry-based analyses [68,69], which have found NP to be most common in early treatment, with incidence decreasing after 18 weeks. These evolving guidelines may reduce the burden of haematological monitoring and may improve the utilisation of clozapine for the treatment of PDP due to changing perspectives on risk, and improved capacity in dedicated clozapine clinics from these reduced monitoring requirements.

A collaborative, multidisciplinary approach involving both movement disorder and mental health specialists is advised when managing PDP, due to its impact on quality of life, morbidity and mortality. Dedicated PDP clozapine services and early involvement of community mental health teams to robustly monitor for PDP may improve long-term outcomes for PD patients.

The strengths of this review include its scope; completing a systematic search for publications investigating the efficacy and tolerability of APs in the treatment of PDP over four scientific databases, including all currently available APs worldwide to reduce the likelihood of arbitrarily disregarding studies completed with APs not available in the UK. Only primary data has been utilised; double-blind, randomised, placebo-controlled studies that have been published in a peer reviewed journal have been included, providing the highest level of evidence for treatment effect.

The limitations of this review include: its restriction of eligible studies to published, peer reviewed double-blind, randomised, placebo-controlled trials, which excluded double-blind comparator studies that may have provided comparative effectiveness data for a wider range of APs. This limits the number of trials and agents included in this review, which may impact its clinical utility. Limiting papers to English language may have also resulted in relevant papers being omitted. Use of automated database search limits may arbitrarily disregard relevant studies, and a lack of preregistration limits the verifiability of a priori review protocol. However, narrowly defined inclusion criteria, restricted to published, peer reviewed double-blind placebo-controlled trials mitigates the risk of selective inclusion exclusion of relevant studies. The exclusion of unpublished trials which may be listed on clinical trial registries may also contribute to publication bias, potentially resulting in data synthesis supporting favourable efficacy or tolerability.

Despite the high prevalence of PDP, there is a paucity of high-quality double-blind, randomised placebo-controlled clinical trials that inform national guidelines, with only four AP medications investigated to this standard of evidence. Among these, inter-study design heterogeneity, as well as limited number of included studies render statistical analysis of provided data more difficult to interpret. Further clinical trials of AP medications, particularly those with low D_2_ binding affinity, may help to improve the evidence base that is currently used to inform national prescribing guidelines. Direct head-to-head pimavanserin clozapine studies may more clearly demonstrate comparative efficacy and tolerability, as well as comparator studies between pimavanserin and quetiapine—the most commonly prescribed AP medication for PDP in the UK [29,32]. Tolerability and long-term safety studies for newer agents such as pimavanserin, as well as the currently NICE recommended clozapine in PD cohorts would be of significant benefit for clinicians to better understand balance of risk when prescribing for their patients.

## Figures and Tables

**Table 1:** Previous reviews of Antipsychotic treatment for PDP.

Paper	Type	Trial Types Included	AP Search
Shotbolt, 2010 [33]	Systematic Review	OL, DBRPCT, COMP	QTP
Jethwa, 2015 [34]	Meta-Analysis	DBRPCT	Any antipsychotic
Iketani, 2017 [38]	Network Meta-Analysis	OL, DBRPCT, COMP	Atypical antipsychotics
Zhang, 2019 [35]	Meta-Analysis	DBRPCT	QTP, OLZ, PIM, CLO
Iketani, 2020 [36]	Network Meta-Analysis	OL, DBRPCT, COMP	Any antipsychotic
Mansuri, 2022 [40]	Meta-Analysis	DBRPCT	PIM
Yunusa, 2023 [39]	Network Meta-Analysis	OL, DBRPCT, COMP	Any antipsychotic
Srisurapanont, 2024 [37]	Network Meta-Analysis	DBRPCT, COMP	Any antipsychotic

Abbreviations—AP—Antipsychotic, CLO—Clozapine, COMP—Comparator, DBRPCT—Double-Blind Randomised Placebo-Controlled Trial, OL—Open Label, OLZ—Olanzapine, PIM—Pimavanserin.

**Table 2:** Characteristics of Included Studies.

Author, Location Antipsychotic	Psychotic Symptom	Duration (Weeks)	Participants					Psychosis Entry Criteria	Dementia Criteria	PD Diagnostic Criteria	Measures	Medication Titration
			Active vs. Control	Age ± SD	Mean PD Duration Years ± SD	M/F	MMSE					
Shotbolt et al., 2009 (UK) [45] Quetiapine	Hallucinations and/or delusions	12	QTP: 11 PLA: 13	74 ± 8 70 ± 8	8 ± 4 9 ± 5	7/4 9/4	24.6 ± 3.6 20.8 ± 5.7	>3/7 on BPRS for Hallucinations, Suspiciousness or unusual thought content. Symptoms present for ≥2 weeks	No exclusions for dementia	UK Brain Bank Criteria	Time to drop out BPRS NPI Baylor Scale UPDRS total UPDRS motor	Week 1 25 mg OD, Week 2 25 mg BD, Week 3 50 mg BD Optional increase to 50 mg am 100 mg pm No reductions allowed
Rabey et al., 2007 (Israel) [46] Quetiapine	Auditory or visual hallucinations and/or delusions	12	QTP: 30 PLA: 28	75.5 ± 8.1 74.5 ± 8.7	10.5 ± 6.4 10.6 ± 6.4	17/13 16/12	22.6 ± 6.0 21.7 ± 6.0	“Severe visual or auditory hallucinations and/or delusions which significantly affected the patient’s quality of life” CGI-S ≥4 for psychosis No duration requirements specified	No exclusions for dementia	UK Brain Bank Criteria	BPRS CGI-S UPDRS	Start dose of 12.5 mg, increasing every 2–3 days until symptoms cleared or side effects limited treatment No reductions allowed
Ondo et al., 2005 (USA) [47] Quetiapine	Visual hallucinations	12	QTP: 21 PLA: 10	74 ± 7 71 ± 5	12 ± 7 9 ± 4	11/10 6/4	26.1 ± 2.5 27.0 ± 2.9	“Subjectively problematic Visual Hallucinations” No duration requirements specified	Participants required to score ≥21 on MMSE for inclusion	No PD criteria specified	BPRS Baylor Scale GDRS UPDRS	Titrated to 50 mg BD over three weeks, then titrated to 100 mg BD over the following 3 weeks Reductions allowed
Fernandez et al., 2009 (USA) [48] Quetiapine	Visual hallucinations	8	QTP: 8 PLA: 8	64.6 ± 7.5 71.5 ± 7.5	Not provided Not provided	Not provided Not provided	Not provided Not provided	“Experiencing consistent and persistent predominantly nocturnal Visual Hallucinations” Symptoms present for ≥1 month	Patients excluded if had “significant cognitive impairment”	UK Brain Bank Criteria	BPRS CGI-S UPDRS	Commenced on 25 mg OD, increased every 3–7 days by 25 mg until final dose of 150 mg OD or complete resolution of symptoms No reductions allowed
Ondo et al., 2002 (USA) [49] Olanzapine	Auditory and visual hallucinations	9	OLZ: 18 PLA: 12	71.0 ± 71 (Pooled)	9.6 ± 5.1 (Pooled)	19/11 (Pooled)	26.8 ± 3.3 (Pooled)	‘Drug induced hallucinations, which were clinically problematic enough to justify intervention” No duration requirements specified	Patients required to score >20 on MMSE for inclusion	“Patients diagnosed with PD based on having 2 of the 3 cardinal criteria (Bradykinesia, rigidity, rest tremor)”	Baylor Scale Finger Tap Test UPDRS	2.5 mg OD for first 3 weeks. with increase by 2.5 mg OD. Maintenance or reduction every 3 weeks. Maximum dose of 10 mg OD Reductions allowed
Breier et al., 2002 (USA) [50] Olanzapine	Hallucinations and/or delusions	4	OLZ: 41 PLA: 42	73.5 ± 8.7 71.7 ± 6.8	12.9 ± ? (SD not provided) 10.6 ± ? (SD not provided)	26/15 32/10	24.0 ± 3.8 24.7 ± 3.9	Individual Hallucinations of Delusions item score of ≥2 on NPI at study entry and randomisation Experienced psychosis in 2 weeks before entry	No exclusions for dementia	DSM-IV criteria for idiopathic PD	BPRS CGI-S NPI UPDRS	2.5 mg OD, with increases of 2.5 mg every 3–4 days up to a maximum of 6 tablets (15 mg) according to clinical response Reductions allowed
Brier et al., 2002 (Europe) [50] Olanzapine	Hallucinations and/or delusions	4	OLZ: 49 PLA: 28	70.9 ± 6.3 70.5 ± 8.2	10.1 ± ? (SD not provided) 15.1 ± ? (SD not provided)	33/16 18/10	24.7 ± 4.8 25.0 ± 3.0	Individual Hallucinations of Delusions item score of ≥2 on NPI at study entry and randomisation Experienced psychosis in 2 weeks before entry	No exclusions for dementia	DSM-IV criteria for idiopathic PD	BPRS CGI-S NPI UPDRS	2.5 mg OD, with increases of 2.5 mg every 3–4 days up to a maximum of 6 tablets (15 mg) according to clinical response Reductions allowed
Parkinson Study Group, 1999 (USA) [51] Clozapine	Hallucinations and/or delusions	4	CLO: 30 PLA: 30	71.9 ± 11.9 70.8 ± 8.6	10.8 ± 6.1 10.4 ± 7.5	20/10 14/15	23.8 ± 4.8 21.7 ± 5.2	“Psychosis severe enough to have warranted treatment with a standard neuroleptic drug if the patient had not had Parkinson’s disease, ≥3 on CGIS for psychosis” Symptoms present for ≥4 weeks	Dementia patients excluded if unable to participate in testing battery. Unclear how this is measured.	≥Three out of four cardinal features (tremor at rest, rigidity, hypokinesia and problems with posture and balance typical of Parkinson’s disease)	BPRS CGI-S SAPS UPDRS	6.25 mg OD increased or decreased every 3 to 4 days until the start of the final week, according to clinical response Reductions allowed
Pollak et al., 2004 (France) [52] Clozapine	Hallucinations and/or delusions	4	CLO: 32 PLA: 28	71.2 ± 7.4 72.8 ± 8.2	12.1 ± 5.7 11.3 ± 5.4	18/14 14/14	26.1 ± 3.0 24.1 ± 2.8	Psychotic symptom score ≥4 for at least one of the items P1 (hallucinations) or P3 (delusions) of the positive sub-score PANSS >3 on CGIS for psychosis Symptoms present for ≥4 weeks	Patients required to score >20 on MMSE for inclusion	UK Brain Bank Criteria	CGIS PANSS UPDRS	6.25 mg OD, increased by 12.5 mg up to three times per week, up to a maximum dose of 50 mg OD Reductions allowed
Cummings et al., 2014 (USA, Canada) [53] Pimavanserin	Hallucinations and/or delusions	6	PIM: 105 PLA: 94	72.4± 6.6 72.4 ± 7.9	Not provided Not provided	64/31* 52/38*	26.0 ± 2.6 26.6 ± 2.4	Combined score of ≥6 or individual score of ≥4 on NPI delusions and/or hallucinations. Symptoms severe enough to warrant antipsychotic treatment Symptoms present for ≥4 weeks	Patients required to score ≥21 on MMSE for inclusion	UK Brain Bank Criteria	SAPS-PD SAPS CGI-S UPDRS	Fixed dose for 40 mg OD No reductions
Meltzer et al., 2010 (USA) [54] Pimavanserin	Hallucinations and/or delusions	4	PIM: 29 PLA: 31	72.3 ± 1.4 69.6 ± 1.7	Not provided Not provided	26/3 20/11	Not provided Not provided	Psychosis severity of ≥4 on NPI for hallucinations and delusions, must have been present for ≥4 weeks	Patients with dementia excluded, no explicit criteria provided	None Specified	SAPS PPRS CGI-S UPDRS	20 mg OD, with titration to 40 mg and 60 mg at days 8 and 15 respectively depending on clinical response Reductions not allowed

Abbreviations: BPRS—Brief Psychiatric Rating Scale, Baylor Scale—Baylor PD Hallucination Scale, BD—Bis in Die (twice daily), CGI-S—Clinical Global Impression Scale, CLO—Clozapine, F—Female, GDRS—Goetz Dyskinesia Rating Scale, M—Male, MMSE—Mini Mental State Examination, NPI—Neuropsychiatric Inventory, OD—Omne in Die (Once daily), OLZ—Olanzapine, PANSS—Positive and Negative Symptom Scale; PD—Parkinson’s disease, PIM—Pimavanserin, PLA—Placebo, PPRS—Parkinson’s Psychosis Rating Scale, QTP—Quetiapine, SD—Standard Deviation, SAPS—Scale for the Assessment of Positive Symptoms, SAPS-PD—Scale for the Assessment of Positive Symptoms in Parkinson’s Disease, UPDRS—Unified Parkinson’s Disease Rating Scale. * Patient demographic data for the Cummings et al., (2014) [53] paper only includes patients in the full analysis set—consisting of all patients who had received ≥1 dose and SAPS baseline and ≥1 SAPS post-baseline.

## Data Availability

Data analysed were obtained from previously published studies that have been cited in the reference list and main body text. Extracted data and calculated effect sizes are included within the manuscript Supplementary Materials.

## Supplementary Materials

The following supporting information can be downloaded at: https://www.mdpi.com/article/10.3390/healthcare14050698/s1, Table S1: Quantitative results data for change in psychosis as reported by included clinical studies; Table S2: Quantitative results data for change in motor symptoms as reported by publications.

## Abbreviations

The following abbreviations are used in this manuscript:

α_1_A	Adrenergic Alpha Receptor Type 1A
α_2_A	Adrenergic Alpha Receptor Type 2A
α_2_C	Adrenergic Alpha Receptor Type 2C
ADL	Activities of Daily Living
AE	Adverse Event
AP	Antipsychotic
BD	Bis in Die
BPRS	Brief Psychiatric Rating Scale
CGI-I	Clinical Global Impression—Improvement
CGI-S	Clinical Global Impression—Severity
CLO	Clozapine
COMP	Comparator Trial
D_2_	Dopamine Receptor Type 2
D_3_	Dopamine Receptor Type 3
DA	Dopamine
DBRPCT	Double Blind, Randomised Placebo Controlled Trial
DLB	Dementia with Lewy Bodies
F	Female
GDRS	Goetz Dyskinesia Rating Scale
H_1_	Histamine Receptor Type 1
Ki	Equilibrium Inhibitor Constant
M	Male
M_1_	Muscarinic Receptor Type 1
mg	Milligram
MMSE	Mini Metal State Examination
NICE	National Institute for Health and Care Excellence
nM	Nanomolar
NP	Neutropenia
NPI	Neuropsychiatric Inventory
nr	No Response
NR	Not Reported
OD	Omne in Die
OL	Open Label
OLZ	Olanzapine
PANSS	Positive and Negative Syndrome Scale
PPRS	Parkinson Psychosis Rating Scale
PD	Parkinson’s Disease
PDP	Parkinson’s Disease Psychosis
PIM	Pimavanserin
PLA	Placebo
QTP	Quetiapine
RCT	Randomised Controlled Trial
SAPS	Scale for the Assessment of Positive Symptoms
SAPS H + D	SAPS total Hallucinations and Delusions domain score
SAPS-PD	Scale for the Assessment of Positive Symptoms in Parkinson’s Disease
SD	Standard Deviation
SE	Standard Error
UPDRS	Unified Parkinson’s Disease Rating Scale
5-HT_1_A	Serotonin Receptor Type 1A
5-HT_2_A	Serotonin Receptor Type 2A
5-HT_2_C	Serotonin Receptor Type 2C
5-HT_6_	Serotonin Receptor Type 6
5-HT_7_	Serotonin Receptor Type 7

## Appendix A

**Table A1:** Effect sizes calculated for studies that provided adequate data.

Study + Medication	Measure	Effect Size	95% Confidence Interval	Magnitude
Fernandez et al., 2009 [48] Quetiapine	BPRS total	0.17	−0.82 to 1.15	Small
	BPRS item 12	−1.23	−2.32 to −0.13	Large
Breier et al., 2002 (USA) [50] Olanzapine	CGI-S	0.17	−0.26 to 0.6	Small
	BPRS positive	−0.03	−0.46 to 0.40	Small
	BPRS total	0.06	−0.38 to 0.49	Small
	NPI	0.06	−0.37 to 0.49	Small
Breier et al., 2002 (Europe) [50] Olanzapine	CGI-S	0.00	−0.46 to 0.46	Small
	BPRS positive	0.15	−0.31 to 0.62	Small
	BPRS total	0.14	−0.32 to 0.61	Small
	NPI	0.18	−0.29 to 0.64	Small
The Parkinson Study Group, 1999 [51] Clozapine	CGI-S	−0.82	−1.37 to −0.26	Large
	BPRS total	−0.91	−1.47 to −0.34	Large
	SAPS	−0.78	−1.33 to −0.22	Medium-Large
Pollak et al., 2004 [52] Clozapine	CGI-S	−0.89	−1.42 to −0.36	Large
	PANSS positive	−1.38	−1.95 to −0.81	Large
Cummings et al., 2014 [53] Pimavanserin	CGI-S	−0.50	−0.79 to −0.21	Medium
	SAPS H + D	−0.48	−0.77 to −0.19	Small to Medium
	SAPS-PD	−0.48	−0.77 to −0.18	Small to Medium

Abbreviations: BPRS—Brief Psychiatric Rating Scale; CGI-S—Clinical Global Impression—Severity; NPI—Neuropsychiatric Inventory; PANSS—Positive and Negative Syndrome Scale, SAPS—Scale for the Assessment of Positive Symptoms; SAPS H + D—SAPS total Hallucinations and Delusions domain score; SAPS-PD—Scale for the Assessment of Positive Symptoms in Parkinson’s Disease; SMD—Standardised Mean Difference.
