# Supplementary material for: Antipsychotic Medications in Parkinson’s Disease Psychosis; A Systematic Review of Double-Blind, Randomised, Placebo-Controlled Trials
Source: Healthcare (Basel). 2026 Mar 9;14(5):698. doi: 10.3390/healthcare14050698 (PMC12984574; doi:10.3390/healthcare14050698)
Supplement: Supplementary file 1 [file healthcare-14-00698-s001.zip › healthcare-4058214-supplementary.pdf]

Supplementary Materials – Table S1. Quantitative results data for change in psychosis as reported by included clinical studies.

Studies	Mean dose of antipsychotic	Psychiatric scale	Active group			Placebo group			LS MD	95% CI	Between group Significance	Effect Size
			Baseline ± SD	Study end ± SD	Mean change	Baseline ± SD	Study end ± SD	Mean change				
Shotbolt et al., 2009 [45]	Quetiapine 72.7 ± 26.1 mg	BPRS total	39.2 ± 8.4	35 ± 6.1	NR	41.5 ± 6.5	39 ± 6.4	NR	NR	NR	"Non-significant"	NR
		NPI	15.4 ± 7.4	8.4 ± 2.9	NR	21.5 ± 11.3	12.2 ± 8.3	NR	NR	NR	"Non-significant"	NR
Rabey et al., 2007 [46]	Quetiapine 119.2 ± 56.4 mg	CGI-S	NR	NR	NR	NR	NR	NR	NR	NR	"Non-significant"	NR
		BPRS total	34.2 ± 5.0	34.0 ± 6.7	NR	36.0 ± 8.8	31.9 ± 8.2	NR	NR	NR	"Non-significant"	NR
Ondo et al., 2005 [47]	Quetiapine 169.1 mg	BPRS total	11.0 ± 5.0	NR	NR	11.0 ± 5.0	NR	NR	NR	NR	"Non-significant"	NR
		Baylor Scale	NR	NR	NR	NR	NR	NR	NR	NR	"Non-significant"	NR
Fernandez et al., 2009 [48]	Quetiapine 58.3 mg	CGI-S	NR	2.76 ± 0.77	NR	NR	3.85 ± 1.01	NR	NR	NR	p=0.03	NR
		BPRS total	31.2 ± 9.43	NR	1.00 ± 6.97	30.2 ± 7.49	NR	-0.28 ± 7.63	NR	NR	"Non-significant"	NR
		BPRS item 12	3.5 ± 1.06	NR	-1.32 ± 1.13	3.3 ± 0.92	NR	-0.04 ± 0.82	NR	NR	p=0.02	NR
Ondo et al., 2002 [49]	OLZ 4.6 ± 2.2 mg	Interview	13.0 ± 4.2	9.5 ± 6.8	NR	13.9 ± 3.3	11.1 ± 4.7	NR	NR	NR	"Non-significant"	NR
Breier et al., 2002 (USA) [50]	Olanzapine 4.2 ± 2.2 mg	CGI-S	4.2 ± 0.8	NR	-0.7 ± 1.1	4.2 ± 0.8	NR	-0.9 ± 1.2	NR	NR	p=0.341	NR
		BPRS positive	6.7 ± 3.2	NR	-1.7 ± 3.5	6.9 ± 4.0	NR	-1.6 ± 3.9	NR	NR	p=0.962	NR
		BPRS total	18.1 ± 10.0	NR	-2.7 ± 8.3	18.2 ± 9.8	NR	-3.1 ± 5.9	NR	NR	p=0.677	NR
		NPI	31.9 ± 20.1	NR	-7.0 ± 20.9	31.1 ± 21.4	NR	-8.1 ± 16.5	NR	NR	p=0.775	NR
Breier et al., 2002 (Europe)[50]	Olanzapine 4.1 ± 2.0 mg	CGI-S	4.0 ± 1.0	NR	-0.8 ± 1.4	4.0 ± 1.3	NR	-0.8 ± 1.5	NR	NR	p=0.993	NR
		BPRS positive	7.5 ± 3.0	NR	-2.3 ± 4.1	8.2 ± 3.5	NR	-2.9 ± 3.4	NR	NR	p=0.612	NR
		BPRS total	17.9 ± 9.0	NR	-4.3 ± 8.3	20.6 ± 9.4	NR	-5.5 ± 8.3	NR	NR	p=0.735	NR
		NPI	25.1 ± 15.6	NR	-6.4 ± 13.1	26.0 ± 15.0	NR	-8.8 ± 13.8	NR	NR	p=0.496	NR
The Parkinson Study Group, 1999 [51]	Clozapine 24.7 mg	CGI-S	4.4 ± 0.8	NR	-1.6 ± 0.3 SE	4.4 ± 1.0	NR	-0.5 ± 0.2 SE	NR	NR	p<0.001	NR
		BPRS total	33.1 ± 9.9	NR	-9.3 ± 1.5 SE	35.0 ± 10.7	NR	-2.6 ± 1.3 SE	NR	NR	p=0.002	NR
		SAPS	20.9 ± 13.0	NR	-11.8 ± 2.0 SE	22.4 ± 12.3	NR	-3.8 ± 1.9 SE	NR	NR	p=0.01	NR
Pollak et al., 2004 [52]	Clozapine 38.5 mg	CGI-S	5.1 ± 0.8	3.3 ± 1.5	-1.8 ± 1.5	4.9 ± 0.9	4.3 ± 1.5	-0.6 ± 1.1	NR	NR	p=0.001	NR
		PANSS positive	17.8 ± 4.7	12.3 ± 4.1	-5.6 ± 3.9	15.3 ± 5.0	14.5 ± 5.7	-0.8 ± 2.8	NR	NR	p<0.0001	NR
Cummings et al., 2014 [53]	Pimavanserin 40 mg	CGI-S	4.27 ± 0.92	NR	-1.02 ± 0.12 SE	4.32 ± 0.91	NR	-0.44 ± 0.12 SE	-0.58	-0.92 to -0.25	p=0.0007	0.52
		CGI-I	-	2.78 ± 0.14 SE	NR	-	3.45 ± 0.14 SE	NR	-0.67	-1.06 to -0.27	p=0.0011	0.51
		SAPS H+D	17.5 ± 7.75	NR	-6.51 ± 0.72 SE	15.8 ± 6.52	NR	-3.14 ± 0.73 SE	-3.37	-5.40 to -1.35	p=0.0012	0.50
		SAPS-PD	15.9 ± 6.12	NR	-5.79 ± 0.66 SE	14.7 ± 5.55	NR	-2.73 ± 0.67 SE	-3.06	-4.91 to -1.20	p=0.0014	0.50
Meltzer et al., 2010 [54]	Pimavanserin 44.8 ± 16 mg	CGI-S	NR	NR	NR	NR	NR	NR	-0.4	-1.10 to 0.20	p=0.2	0.58
		SAPS H+D	16.7 ± 7.45	11.0 ± 11.09	NR	17.9 ± 11.79	16.8 ± 14.35	NR	-4.6	-10.0 to 0.70	p=0.09	0.56
		SAPS Rated H+D	5.4 ± 1.76	3.5 ± 2.84	NR	5.3 ± 2.54	5.1 ± 3.06	NR	-1.89	-3.39 to -0.39	p=0.02	0.66
		PPRS	NR	NR	NR	NR	NR	NR	-1.2	-2.60 to 0.30	p=0.11	0.48

Abbreviations: CGI-S – Clinical Global impression Severity; BPRS – Brief Psychiatric Rating Scale; Baylor – Baylor Hallucination Scale; Interview – Structured interview for hallucinations in PD; CI – Confidence Interval; LS MD– Least Squares Mean Difference; mg- milligram, NR – Not Reported; NS – Non-significant; NPI – Neuropsychiatric Inventory; PANSS – Positive and Negative syndrome scale; PPRS – Parkinson Psychosis Rating Scale; SAPS – Scale for the Assessment of Positive Symptoms; SAPS-PD – Scale for the Assessment of Positive Symptoms in Parkinson's Disease; SAPS H+D – SAPS total Hallucinations and Delusions domain score; SAPS Rated H+D – SAPS global ratings total Hallucinations + Delusions score; SD – Standard Deviation; SE – Standard Error.

Negative figures represent improvement

Table S2. Quantitative results data for change in motor symptoms as reported by publications

Studies	Antipsychotic	Motor scale	Active group			Placebo group			LS MD	95% CI	Between group Significance	Effect Size
			Baseline ± SD	Study end ± SD	Mean change	Baseline ± SD	Study end ± SD	Mean change				
Shotbolt et al., 2009 [45]	Quetiapine	UPDRS motor	31.2 ± 14.1	28.2 ± 12.3	NR	29.0 ± 16.8	30.1 ± 10.4	NR	NR	NR	"Non-significant"	NR
Rabey et al., 2007 [46]	Quetiapine	UPDRS motor	37.0 ± 9.6	39.2 ± 9.8	NR	39.5 ± 13.1	37.6 ± 14.7	NR	NR	NR	"Non-significant"	NR
Ondo et al., 2005 [47]	Quetiapine	UPDRS motor	34.0 ± 8.0	NR*	NR	30.70 ± 11.9	NR*	NR	NR	NR	"Non-significant"	NR
		GDRS†	2.0 ± 3.3	NR	NR	5.6 ± 5.2	NR	NR	NR	NR	"Non-significant"	NR
Fernandez et al., 2009 [48]	Quetiapine	UPDRS motor	31.2 ± 9.43	25.46 ± 6.84	NR	30.2 ± 7.49	33.03 ± 7.46	NR	NR	NR	"Non-significant"	NR
Ondo et al., 2002 [49]	Olanzapine	UPDRS motor	NR	NR	4.6 ± 6.9	NR	NR	NR	NR	NR	p<0.05	NR
		Finger Tap Test	NR	NR	NR	NR	NR	NR	NR	NR	p<0.05	NR
Breier et al., 2002 (USA) [50]	Olanzapine	UPDRS motor	20.5 ± 7.4	NR	2.6 ± 6.0	20.6 ± 9.1	NR	-0.2 ± 4.3	NR	NR	p=0.023	NR
		CGIS-S motor	4.1 ± 0.7	NR	0.3 ± 0.7	4.1 ± 0.8	NR	-0.2 ± 0.5	NR	NR	p<0.001	NR
Breier et al., 2002 (Europe) [50]	Olanzapine	UPDRS motor	18.7 ± 7.2	NR	2.7 ± 6.0	21.3 ± 7.2	NR	-0.3 ± 5.0	NR	NR	p=0.039	NR
		CGIS-S motor	4.1 ± 0.8	NR	0.3 ± 0.8	4.1 ± 1.1	NR	0.1 ± 0.7	NR	NR	p=0.261	NR
Parkinson Study Group, 1999 [51]	Clozapine	UPDRS motor	32.8 ± 11.3	NR	-3.6 ± 1.9 SE	37.1 ± 13.0	NR	-1.8 ± 1.2 SE	NR	NR	p=0.34	NR
Pollak et al., 2004 [52]	Clozapine	UPDRS motor	31.5 ± 14.2	NR	-3.5 ± 7.7	31.4 ± 13.2	NR	-3.0 ± 8.1	NR	NR	p=0.85	NR
Cummings et al., 2014 [53]	Pimavanserin	UPDRS motor	32.8 ± 12.86	NR	-0.86‡	33.3 ± 12.23	NR	-0.86‡	NR	NR	"Non-significant"	NR
Meltzer et al., 2010 [54]	Pimavanserin	UPDRS motor	NR	NR	-0.99‡	NR	NR	-2.64‡	1.65	-2.25 to 5.55	p=0.4	NR

Abbreviations: GDRS – Goetz Dyskinesia Rating Scale; SD – Standard Deviation; SE – Standard Error; LS MD – Least Squares Mean Difference; NR – Not Reported; UPDRS – Unified Parkinson's Disease Rating Scale.

Negative numbers represent improvement in all measures included in table.

* - Post treatment results displayed in graph format, with inadequate data for inclusion. † - GDRS between groups was significantly different at baseline. ‡ SD/SE not provi
